# Spices in a High-Saturated-Fat, High-Carbohydrate Meal Reduce Postprandial Proinflammatory Cytokine Secretion in Men with Overweight or Obesity: A 3-Period, Crossover, Randomized Controlled Trial

**DOI:** 10.1093/jn/nxaa063

**Published:** 2020-03-25

**Authors:** Ester S Oh, Kristina S Petersen, Penny M Kris-Etherton, Connie J Rogers

**Affiliations:** 1 Department of Nutritional Sciences, The Pennsylvania State University, University Park, PA, USA; 2 Center for Molecular Immunology and Infectious Disease, The Pennsylvania State University, University Park, PA, USA

**Keywords:** monocytes, inflammatory cytokines, obesity, nutritional intervention, randomized controlled trial

## Abstract

**Background:**

Postprandial inflammation that occurs concurrently with hyperglycemia and hyperlipidemia after ingestion of a high-saturated-fat, high-carbohydrate meal (HFCM) is a risk factor for cardiovascular disease (CVD). Numerous preclinical and clinical studies demonstrate anti-inflammatory effects of individual spices. However, the effect of consumption of a spice blend on inflammatory mediators has not been examined in a randomized controlled trial.

**Objectives:**

The objective of this study was to investigate the postprandial effect of a blend of spices in a HFCM on inflammatory cytokine responses.

**Methods:**

Nonsmoking men (40–65 y old) with overweight/obesity (25 ≤ BMI ≤ 35 kg/m^2^), elevated waist circumference (≥ 94 cm), and ≥ 1 CVD risk factor were recruited for a 3-period crossover study ( *n* = 12). In random order, participants consumed the following: a HFCM (∼1000 kcal, 33% kcal from saturated fat and 36% kcal from carbohydrate), a HFCM containing 2 g spice blend, or an HFCM containing 6 g spice blend. The spice blend consisted of basil, bay leaf, black pepper, cinnamon, coriander, cumin, ginger, oregano, parsley, red pepper, rosemary, thyme, and turmeric. Blood was collected before, and hourly for 4 h after the HFCM. Peripheral blood mononuclear cells (PBMCs) were isolated, and the percentage of CD14 ^+^/Human Leukocyte Antigen-DR isotype ^+^ (HLA-DR ^+^) monocytes and proinflammatory cytokine concentrations in plasma and LPS-stimulated PBMCs were quantified as secondary outcomes.

**Results:**

There was a significant spice-by-time interaction on IL-1β (*P* < 0.001), IL-8 (*P* = 0.020), and TNF-α (*P =* 0.009) secretion from LPS-stimulated PBMCs. IL-1β secretion from LPS-stimulated PBMCs was significantly reduced (1314%) at 240 min after HFCM consumption containing 6 g, but not 2 g, of spice blend compared with 0 g spice blend.

**Conclusions:**

A HFCM containing 6 g spice blend attenuated HFCM-induced postprandial IL-1β secretion in men with overweight/obesity.

This trial was registered at clinicaltrials.gov as NCT03064958.

## Introduction

Overweight or obesity affects nearly 72% of adults in the United States, and the prevalence of obesity has nearly tripled since 1962 (1). There is a strong association between overweight/obesity and the incidence of cardiovascular disease (CVD) and other metabolic diseases (2), which may be mediated, at least in part, by chronic low-grade inflammation (3). In US adults, the risk of cardiometabolic disease is strongly associated with unhealthy dietary habits (e.g., high intake of saturated fats, refined carbohydrates, and sugar-sweetened beverages, and low intake of whole grains, fruits, and vegetables) (4), which may contribute to elevated postprandial inflammation (5). Postprandial hyperglycemia and hyperlipidemia, which are identified as independent CVD risk factors, induce postprandial inflammation (6). Furthermore, postprandial inflammation is associated with increased risk of CVD (5). Thus, gaining a better understanding of the relation between postprandial hyperglycemia, hyperlipidemia, and inflammation has important clinical implications.

In the postprandial state, increased glucose and free fatty acids enter the tricarboxylic acid (TCA) cycle, overwhelm the oxidative phosphorylation capacity of mitochondria, and lead to oxidative stress that further activates inflammatory pathways (7, 8). Repetitive and prolonged inflammation increases the risk of CVD, which is mediated by several cytokines including IL-1β, IL-6, IL-8, monocyte chemoattractant protein (MCP)-1, and TNF-α. IL-1β, MCP-1, and TNF-α increase the expression of adhesion molecules on vascular endothelial cells and serve as chemoattractants, inducing the recruitment of inflammatory cells to the intima of blood vessels (9, 10). IL-6 and IL-8 are involved in leukocyte trafficking across the vascular endothelium (11, 12), contributing to vascular inflammation and atherosclerotic plaque formation (13, 14). Postprandial inflammation and oxidative damage are augmented in a state of metabolic dysfunction (e.g., obesity and type 2 diabetes) (15–18). Furthermore, chronic low-grade inflammation may be exacerbated by postprandial inflammation (19). Thus, the identification of novel dietary strategies to reduce postprandial inflammation is of public health importance given the prevalence of chronic low-grade inflammation and cardiometabolic diseases in the population.

Spices not only add flavor to food, but also may confer various health benefits likely due to the presence of bioactive compounds (20). Numerous preclinical and clinical studies demonstrate anti-inflammatory effects of individual spices and their bioactive compounds such as turmeric (curcumin), ginger (gingerol), and cinnamon (cinnamaldehyde). However, the effect of the consumption of a spice blend, as typically occurs in a meal, on inflammatory mediators has not been examined in humans in a randomized controlled trial.

Therefore, the purpose of this study was to examine the effect of consuming a spice blend in a standardized high-saturated-fat, high-carbohydrate test meal compared with the same meal without the spice blend, on postprandial inflammation in middle-aged men with overweight/obesity. Similar to previous research in this area (21–23), we chose to recruit a small number of homogeneous subjects, and used a crossover design to minimize interpersonal variability in order to ascertain whether spice consumption induced an anti-inflammatory effect in the postprandial setting.

## Methods

### Subjects

We recruited 12 nonsmoking men (40–65 y old) with overweight or obesity (25 ≤ BMI ≤ 35 kg/m ^2^), elevated waist circumference (≥94 cm), and 1 of the following CVD risk factors: altered lipid profile [LDL cholesterol >130 mg/dL, triglyceride (TG) ≥150 mg/dL, or HDL cholesterol <40 mg/dL], elevated C-reactive protein (CRP) (>1 mg/dL), elevated blood pressure [systolic blood pressure (SBP) ≥130 mm Hg and DBP ≥85 mm Hg], or elevated fasting glucose (≥100 mg/dL). Exclusion criteria included having diabetes (fasting glucose >126 mg/dL) or hypertension (SBP >160 mm Hg or DBP >100 mm Hg); receiving any antihypertensive or glucose-lowering drugs; having established CVD, stroke, diabetes, liver, kidney, or autoimmune disease; using cholesterol/lipid-lowering medication or supplements (psyllium, fish oil, soy lecithin, and phytoestrogens) and botanicals; and having weight loss of ≥10% body weight within the 6 mo before enrolling in the study. We originally planned to enroll 6 participants; however, a preliminary analysis revealed a trend toward a different postprandial inflammatory response based on fasting blood glucose concentration (normal fasting glucose range: <100 mg/dL, compared with impaired fasting glucose range: ≥100 mg/dL). Thus, we enrolled 7 additional participants with the goal of having half of the participants with normal fasting glucose and the remainder with impaired fasting glucose. One participant was excluded from the analysis because we failed to collect peripheral blood mononuclear cells (PBMCs) from the participant.

### Recruitment, screening, and random assignment

Participants were recruited through advertisements using flyers on campus and in the community. Individuals who were on the university email lists and who had previously participated in other studies were contacted. They were given further information about the study and screened for eligibility by telephone if interested. Potential participants were scheduled for a screening visit at the Clinical Research Center on the Pennsylvania State University-University Park campus. Participants fasted for 12 h and abstained from alcohol for 48 h before the screening visit. Height, weight, blood pressure, and waist circumference were measured. A blood sample was collected and sent to Quest Diagnostics (Pittsburgh, PA) for biochemical measurement of glucose, lipid profile, and CRP. Eligible participants (*n* = 12) were randomly assigned to treatment sequences using a computer-generated scheme (www.randomization.com). The randomization code was kept by the kitchen staff preparing the meals and the code was broken at the end of the study.

### Study design

The study was a 3-period, crossover, randomized controlled trial (NCT03064958) that was designed to assess the effect of spice consumption on serum TGs as the primary outcome. The study was powered to detect a change in TGs between the treatment groups, and 13 subjects were estimated to yield a TG difference of 15 ± 32 mg/dL (mean ± SD) with 80% power (α = 0.05). These results are summarized in a separate manuscript (under review). We quantified inflammatory responses in 12 subjects because we could not collect enough blood from 1 subject to perform the assays needed to assess inflammatory cytokine secretion in cultured PBMCs. In random order, participants consumed the following meals: *1*) a ∼1000-kcal high-saturated-fat, high-carbohydrate meal (HFCM) containing 33% kcal from saturated fat and 36% kcal from carbohydrate with no additional spices; *2*) a HFCM containing 2 g spice blend; or *3*) a HFCM containing 6 g spice blend, with a ≥3-d washout period between intervention days. The nutrient profile of the HFCM and composition of the spice blend in the HFCM are presented in **Tables 1** and **2**, respectively. The doses of spices used in the current study were chosen to incorporate doses on the low and high ends of daily spice consumption per capita in the United States in 2015 (mean 4.5 g/d) (24). The spices were chosen based on previous studies that reported benefits on CVD and inflammatory outcomes (25–28). In addition, the spices chosen are among the most widely consumed spices in the US diet (29). The test meal was coconut chicken curry, a corn muffin, and a cinnamon biscuit. All the experiments in this study were performed with the approval of the Institutional Review Board of the Pennsylvania State University-University Park campus.

**TABLE 1:** Nutrient profile of the high-saturated-fat, high-carbohydrate meal^1^

Nutrient profile	Coconut chicken curry	Corn muffin	Cinnamon biscuit	Total
Energy, kcal	617	354	105	1076
Total fat, g	39.7	15.8	4.0	59.5
Total fat, % kcal	33.2	13.2	3.3	49.8
Saturated fat, g	32.5	5.7	1.1	39.3
Saturated fat, % kcal	27.2	4.8	0.9	32.9
Carbohydrates, g	40.4	41.3	16.1	97.7
Carbohydrates, % kcal	15.0	15.4	6.0	36.3
Protein, g	26.6	14.9	1.4	42.9
Protein, % kcal	9.9	5.5	0.5	15.9
Dietary fiber, g	0.4	1.3	1.3	2.9

1 Nutrient values were determined using the Nutrient Data System for Research (Minneapolis, MN).

**TABLE 2:** Composition of spice blend in the high-saturated-fat, high-carbohydrate meal

	2 g spice blend				6 g spice blend			
Spice	Coconut chicken curry	Corn muffin	Cinnamon biscuit	Total	Coconut chicken curry	Corn muffin	Cinnamon biscuit	Total
Turmeric, g	0.35	—	—	0.35	1.05	—	—	1.05
Ginger, g	0.13	—	0.13	0.26	0.38	—	0.38	0.76
Cinnamon, g	0.08	—	0.15	0.23	0.23	—	0.45	0.68
Oregano, g	—	0.19	—	0.19	—	0.56	—	0.56
Parsley, g	—	0.14	—	0.14	—	0.41	—	0.41
Basil, g	—	0.13	—	0.13	—	0.40	—	0.40
Coriander, g	0.13	—	—	0.13	0.40	—	—	0.40
Cumin, g	0.13	—	—	0.13	0.40	—	—	0.40
Red pepper, g	0.13	—	—	0.13	0.40	—	—	0.40
Rosemary, g	—	0.10	—	0.10	—	0.31	—	0.31
Black pepper, g	0.08	—	—	0.08	0.23	—	—	0.23
Bay leaf, g	—	0.06	—	0.06	—	0.20	—	0.20
Thyme, g	0.06	—	—	0.06	0.20	—	—	0.20
Total, g	—	—	—	2.00	—	—	—	6.00

### Blood sample collection

A baseline blood sample was collected in sterile EDTA (K2)-coated blood tubes (BD Biosciences), and participants were asked to consume the test meal within 15 min. Blood samples were collected at timed intervals (60, 120, 180, and 240 min) after meal consumption. No other foods or drinks (other than water) were allowed for the remainder of the 240-min testing period. Blood samples were centrifuged at 1800 × *g* for 15 min at room temperature. Plasma was dispensed into microcentrifuge tubes and frozen at −80°C until analysis.

### Proinflammatory cytokine secretion assay

PBMCs were isolated from blood as previously described (30). PBMCs (2 × 10^5^/mL) were stimulated with 0.625 μg/mL LPS (Sigma-Aldrich) in round-bottomed 96-well plates, and supernatants were harvested after 4 h incubation and frozen at −80°C until analysis.

### Measurement of proinflammatory cytokine concentrations

Cytokines and chemokines (IFN-γ, IL-1β, IL-2, IL-4, IL-6, IL-8, IL-10, IL-12p70, IL-13, TNF-α, and MCP-1) in plasma and supernatants were measured using the V-PLEX Proinflammatory Panel 1 Human Kit and V-PLEX Human MCP-1 kit (Meso Scale Diagnostics) as per the manufacturers’ instructions. For the data points that were below the detection range, half of the lower limit of detection was used as a value in the analyses.

### Flow cytometric analysis

**FIGURE 1:**
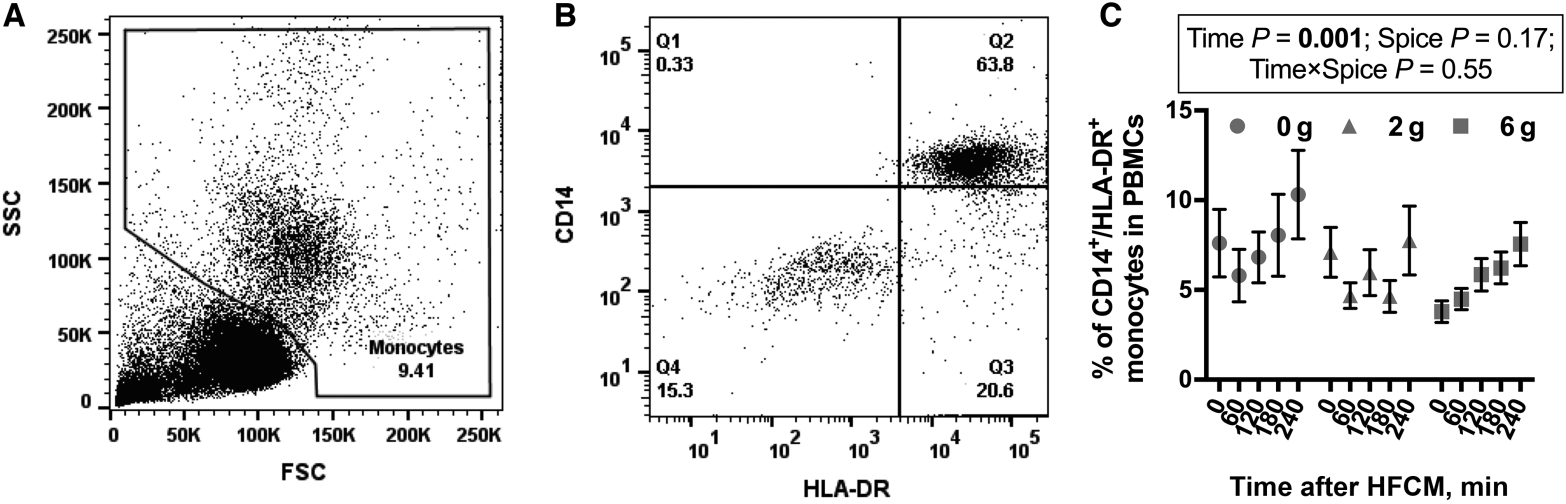
The percentage of monocytes in circulation after an HFCM challenge containing 0 g, 2 g, or 6 g spice blend in men with overweight or obesity at risk of cardiovascular disease. (A) Representative dot plot of FSC against SSC and gate of the monocyte population. (B) Representative dot plot of Human Leukocyte Antigen-DR isotype (HLA-DR) against CD14 expression on cells in the monocyte gate. (C) The percentage of CD14^+^/HLA-DR^+^ monocytes. Data are mean ± SEM. *n* = 7. FSC, forward scatter; HFCM, high-saturated-fat, high-carbohydrate meal; PBMC, peripheral blood mononuclear cell; SSC, side scatter.

PBMCs were stained with fluorescently labeled antibodies as previously described (30). Antibodies for immune cell markers included CD3, CD4, CD8, CD14, CD19, CD56, and Human Leukocyte Antigen-DR isotype (HLA-DR). Antibody isotype controls included mouse IgG_2b_, mouse IgG_2a_, and mouse IgM. CD56 was purchased from BD Biosciences and all remaining antibodies were purchased from BioLegend. A total of 50,000 events were acquired with BD LSR-Fortessa (BD Biosciences). Data were analyzed and plotted using FlowJo 10 (FlowJo, LLC). The monocyte population within the total PBMC population was gated based on forward scatter and side scatter (**Figure 1**A). The percentage of CD14^+^/HLA-DR^+^ cells in the monocyte gate was quantified per sample (Figure 1B) and the percentage of CD14^+^/HLA-DR^+^ monocytes within PBMCs was calculated. We could not collect enough blood in 5 subjects to run flow cytometric analyses; thus, we assessed the percentage of CD14^+^/HLA-DR^+^ in only 7 subjects.

### Statistical analyses

Statistical analyses were performed using SAS version 9.4 (SAS Institute). The percentage of CD14^+^/HLA-DR^+^ monocytes and inflammatory cytokine concentrations were secondary outcomes in the study design. Normality of the data was confirmed by a Q–Q plot assessment. Equal variance was confirmed by residual-versus-predicted value plots. Plasma cytokine concentration and cytokine secretion from PBMCs are reported as the difference between baseline and individual time points after meal consumption. Data points that were >3 SDs from the mean were considered as outliers and removed. A mixed-effects model for repeated measures was used to test the effects of time and/or treatment (spice) on the distribution of immune cell populations (monocytes, CD4^+^ T cells, CD8^+^ T cells, B cells, and NK cells) and postprandial proinflammatory cytokine concentrations in the plasma and in culture supernatants (**Supplemental Table 1**). Outcomes were modeled as repeated measures with a first-order autoregressive matrix. For postprandial cytokine secretion after the HFCM challenge, plasma glucose and its interaction with treatment (spice) were included as covariates because the change in plasma glucose was associated with changes in cytokine secretion. Baseline outcomes were not included as covariates because the models that included baseline outcome as a covariate were not statistically different from models that did not. Participant was designated as a random factor, and the treatment (spice), time, and covariates were fixed factors. In cases where significant treatment (spice) effects or time-by-treatment (spice) interactions were detected, separate analyses were performed to determine between–treatment group (spice) effects at each time point, and time effects for each treatment (spice) group, using mixed-effects analyses for repeated measures followed by Tukey's post hoc test. For all outcomes, α was set as 0.05 and statistical significance was accepted at *P* < 0.05. Graphs were plotted using Prism 7 (GraphPad). Values are reported as mean ± SEM.

## Results

### Baseline characteristics

**Table 3** presents anthropometric measurements, blood pressure, and biochemical measurements at screening. The participants were middle-aged nonsmoking men (51.8 ± 2.7 y) with overweight/obesity (BMI 29.4 ± 0.7 kg/m^2^) and elevated waist circumferences (100.1 ± 1.3 cm). There was a wide range in blood pressure and biochemical measures among participants because only 1 additional risk factor for CVD was required as per the inclusion criteria.

**TABLE 3:** Baseline characteristics of participants with overweight or obesity at risk of cardiovascular disease^1^

Characteristic	Participants
Age, y	51.8 ± 2.7 (40–64)
BMI, kg/m^2^	29.4 ± 0.7 (25.9–33.8)
Waist circumference, cm	100 ± 1.3 (94.2–101)
Blood pressure, mm Hg	
Systolic	122 ± 3.3 (106–138)
Diastolic	78.2 ± 1.6 (70–87)
Glucose, mg/dL	97.1 ± 3.5 (75.0–112)
Total cholesterol, mg/dL	200 ± 10.4 (149–269)
HDL cholesterol, mg/dL	45.0 ± 2.6 (34.0–64.0)
LDL cholesterol, mg/dL	131 ± 8.5 (88.0–195)
Triglycerides, mg/dL	121 ± 16.2 (51.0–202)
C-reactive protein, mg/dL	1.3 ± 0.3 (0.3–3.5)

1 *n* = 12. Values are mean ± SEM (range).

### HFCM challenge increased the percentage of CD14^+^/HLA-DR^+^ monocytes

There was a significant postprandial increase in the percentage of CD14^+^/HLA-DR^+^ monocytes (main effect of time, *P* = 0.001) (Figure 1C) after the HFCM challenge. However, there was no effect of spice and no time-by-spice interaction on the percentage of CD14^+^/HLA-DR^+^ monocytes in circulation (Figure 1C). The percentage of monocytes in circulation was significantly higher at 240 min than at 0, 60, 120, and 180 min after the HFCM challenge (Tukey's post hoc test, *P* < 0.05). The percentages of CD3^+^/CD4^+^ T cells, CD3^+^/CD8^+^ T cells, and CD3^−^/CD56^+^ NK cells were not affected by time or spice (Supplemental Figure 1A, B, C). There was a significant postprandial decrease in the percentage of B cells (main effect of time, *P* = 0.016), but no effect of spice or a time-by-spice interaction on the percentage of CD19^+^ B cells. The percentage of B cells was significantly lower at 180 min than at 0, 60, 120, or 240 min after the HFCM challenge (Tukey's post hoc test, *P* < 0.05) (Supplemental Figure 1D).

### Consumption of a HFCM containing a spice blend attenuated postprandial inflammation

The HFCM challenge alone induced postprandial inflammation, as evidenced by a significant increase in plasma IL-6 concentration and IL-1β secretion from LPS-stimulated PBMCs (data not shown).

**FIGURE 2:**
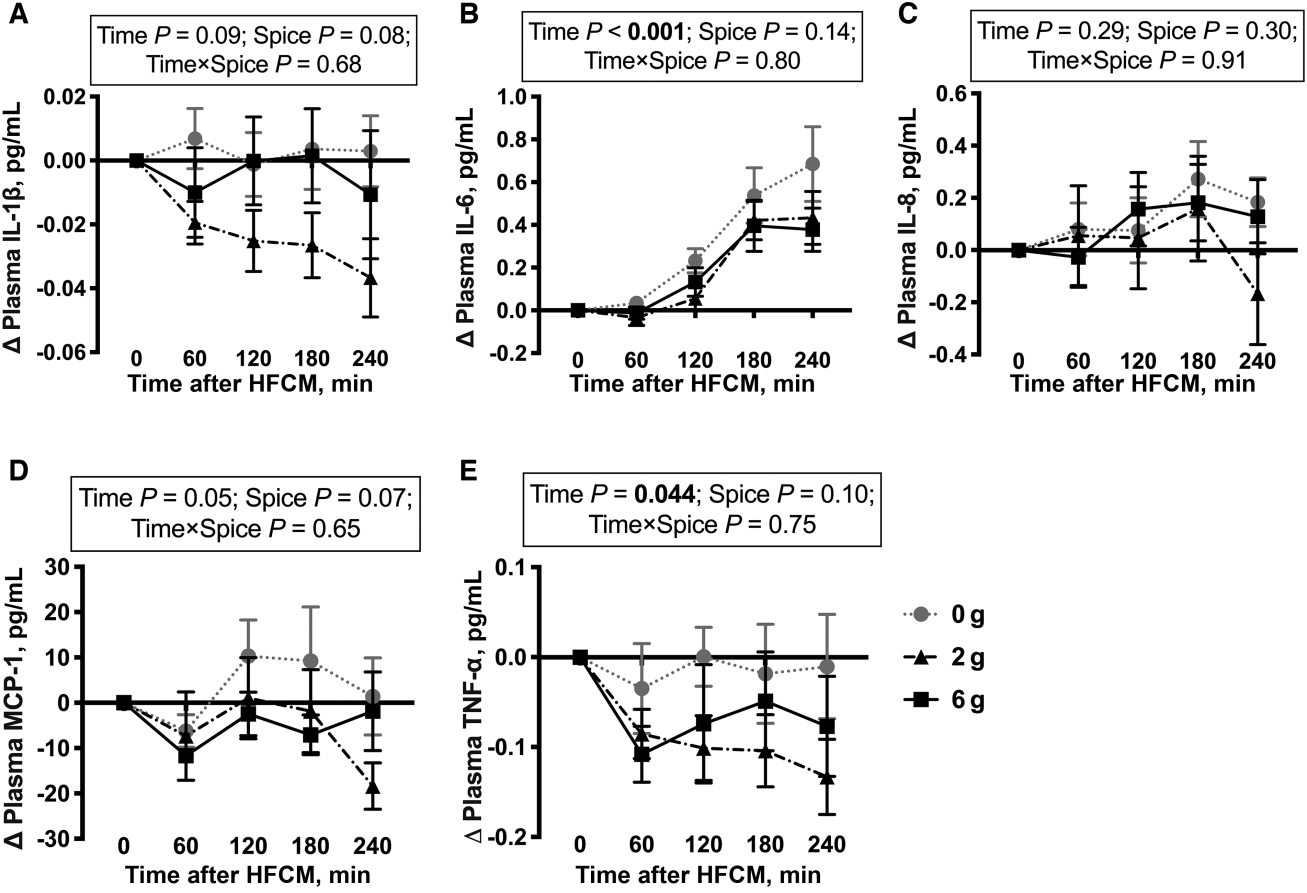
Change in plasma IL-1β (A), IL-6 (B), IL-8 (C), MCP-1 (D), and TNF-α (E) concentrations after a HFCM challenge containing 0 g, 2 g, or 6 g spice blend in men with overweight or obesity at risk of cardiovascular disease. Data are mean ± SEM. *n* = 12. HFCM, high-saturated-fat, high-carbohydrate meal; MCP, monocyte chemoattractant protein.

There was no significant effect of time on plasma IL-1β, IL-8, and MCP-1 (**Figure 2**A, C, D) after HFCM consumption. There was a significant effect of time on plasma concentrations of IL-6 (*P* < 0.001) and TNF-α (*P* = 0.044) (Figure 2B, E). There was no significant spice effect or time-by-spice interaction on plasma IL-1β, IL-6, IL-8, MCP-1, and TNF-α concentrations after HFCM consumption (Figure 2A–E).

**FIGURE 3:**
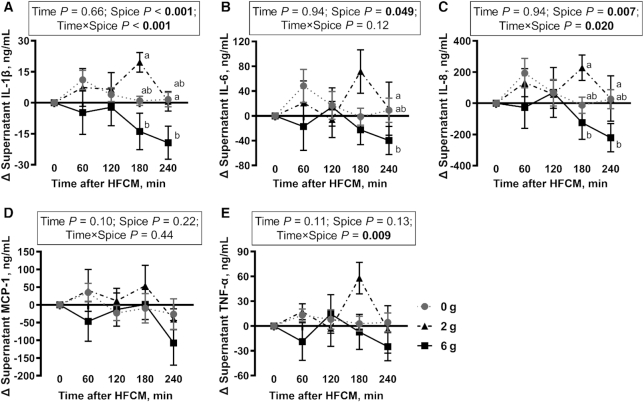
Change in IL-1β (A), IL-6 (B), IL-8 (C), MCP-1 (D), and TNF-α (E) secretion from LPS-stimulated peripheral blood mononuclear cells after a HFCM challenge containing 0 g, 2 g, or 6 g spice blend in men with overweight or obesity at risk of cardiovascular disease. Labeled means at each time point without a common letter differ, *P* < 0.05. Data are mean ± SEM. *n* = 12. HFCM, high-saturated-fat, high-carbohydrate meal; MCP, monocyte chemoattractant protein.

There was no significant overall effect of time on IL-1β, IL-6, IL-8, MCP-1, and TNF-α secretion from LPS-stimulated PBMCs (**Figure 3**A–E). There was a significant spice effect on IL-1β (*P* < 0.001), IL-6 (*P* = 0.049), and IL-8 secretion (*P* = 0.007) after the meal challenge (Figure 3A–C), but no spice effect on MCP-1 and TNF-α secretion (Figure 3D, E). A significant time-by-spice interaction was observed for IL-1β (*P* < 0.001), IL-8 (*P* = 0.020), and TNF-α secretion (*P* = 0.009) after the meal consumption (Figure 3A, C, E), resulting in spice-induced reduction in these cytokines. There was no time-by-spice interaction for IL-6 and MCP-1 secretion (Figure 3B, D).

When the effect of time was analyzed in each treatment group, IL-1β secretion from cultured PBMCs was significantly increased 60 min after HFCM consumption containing 0 g spice blend compared with 0, 120, 180, and 240 min after the meal (Tukey's post hoc test, *P* < 0.05) (**Supplemental Table 2**). IL-1β secretion was significantly reduced at 240 min after HFCM consumption containing 2 g spice blend compared with 0, 60, 120, and 180 min after the meal (Tukey's post hoc test, *P* < 0.05) (Supplemental Table 2). There was no effect of time on IL-β secretion after HFCM consumption containing 6 g spice blend. However, all cytokine change scores were negative after the meal, indicating a decrease from baseline after the meal containing 6 g spice blend (Supplemental Table 2).

When the effect of spice was compared at each time point, IL-1β and IL-8 secretion from cultured PBMCs was significantly lower at 180 min after HFCM consumption containing 6 g spice blend, than for 2 g spice blend, by 171% and 155%, respectively (Figure 3A, C). At 240 min after HFCM consumption containing 6 g spice blend, IL-1β secretion was significantly reduced (1314%) compared with 0 g spice blend (Figure 3A). In addition, at 240 min after HFCM consumption containing 6 g spice blend, IL-6 and IL-8 secretion were significantly lower than for 2 g spice blend, by 445% and 829%, respectively (Figure 3B, C).

There was an effect of spice consumption on plasma IFN-γ (*P* < 0.001), IL-2 (*P* < 0.001), IL-4 (*P* = 0.043), and IL-10 concentrations (*P* = 0.034) (**Supplemental Table 3**) and on IL-2 secretion from LPS-stimulated PBMCs (*P* = 0.040) (**Supplemental Table 4**).

## Discussion

In the current study, we demonstrated that the percentage of monocytes significantly increased in the circulation at 240 min after the HFCM challenge. Furthermore, we demonstrated a significant spice effect on IL-6, and a significant spice-by-time interaction on IL-1β, IL-8, and TNF-α secretion from LPS-stimulated PBMCs. Consumption of 6 g spice blend significantly reduced IL-1β secretion at 240 min after HFCM consumption compared with 0 g spice blend. Furthermore, the change in IL-6, IL-8, MCP-1, and TNF-α secretion from LPS-stimulated PBMCs at 240 min after HFCM consumption containing 6 g spice blend was below baseline values for each cytokine. These results suggest that spice consumption may reduce HFCM-induced postprandial inflammation.

Anti-inflammatory effects of spices and their bioactive compounds are well documented in preclinical and clinical studies. Turmeric is the most commonly used spice in the world. Curcumin, the main bioactive compound in turmeric, alleviates oxidative stress in LPS-injected BALB/c mice and in mouse peritoneal macrophages (31), and reduces IL-1β and TNF-α secretion from LPS-stimulated human monocyte cell lines (32). Curcumin reduces serum inflammatory cytokine concentrations in adults with obesity (33) and metabolic syndrome (34). Ginger has a long history of medical use, some of which is based on its broad anti-inflammatory function. Supplementation with ginger reduces circulating inflammatory markers in subjects with type 2 diabetes (35, 36) and nonalcoholic fatty liver disease (37). Cinnamon exhibits similar anti-inflammatory and immunomodulatory properties in both *in vitro* and *in vivo* animal studies (38, 39), and this is likely due to the action of the bioactive compound cinnamaldehyde (40). In addition, other spices included in our test meal from the current study, such as basil, bay leaf, black pepper, coriander, cumin, red pepper, rosemary, and thyme, may have anti-inflammatory effects (20). Thus, the use of a combination of spices may reduce inflammatory mediators to a greater degree than any individual spice owing to possible differences in bioactivity, duration of effect, and mechanism of action of the individual spices in the blend. Although numerous individual spices show anti-inflammatory properties *in vitro* and *in vivo* in animal models and humans, few studies have examined the effect of the consumption of a spice blend in humans in a randomized controlled trial. Also, few studies have investigated the anti-inflammatory effect of spices in the context of daily meal consumption, which is the typical vehicle of spice consumption. Thus, we developed a test meal containing a spice blend in order to investigate the anti-inflammatory effect of spice in the context of meal consumption. In addition, the spices were selected from the most widely consumed spices in the United States at doses that may be consumed (29), thus representing a blend of spices that may be consumed in the average American diet.

Postprandial inflammation is the response of immune cells, such as monocytes, dendritic cells, and lymphocytes, to the acute postprandial overload of macronutrients that culminates in oxidative stress and inflammation. Metabolism of high amounts of glucose and free fatty acids results in excessive acetyl-CoA production, mitochondrial metabolism of acetyl-CoA via the TCA cycle, and an increase in reactive oxygen species (ROS) within immune cells. This change in the redox status can activate redox-sensitive transcription factors, including NF-κB, which triggers an inflammatory cascade resulting in cytokine secretion (7, 8). There are nutrient-independent and -dependent factors that influence postprandial inflammation. Nutrient-independent factors include obesity, type 2 diabetes, and a sedentary lifestyle, which are associated with chronic low-grade inflammation. Individuals with metabolic disorders such as obesity and diabetes have an augmented postprandial inflammatory response in addition to the elevated inflammatory mediators in the circulation in the fasting state when compared with healthy controls (15). Patel et al. (17) demonstrate prolonged ROS generation and greater intranuclear NF-κB binding activity in PBMCs after high-fat, high-carbohydrate meal consumption in subjects with obesity compared with healthy subjects. Nutrient-dependent factors of postprandial inflammation include caloric content, glycemic index, and lipid profile of the meal (41). Thus, high-fat and/or high-carbohydrate challenge tests are often used to study postprandial inflammation and the effectiveness of nutritional interventions in reducing postprandial inflammation. An acute homeostatic perturbation, such as an HFCM challenge, can be used to capture small changes in inflammatory mediators in the circulation that are not easily observed by static homeostatic measures (42). In the current study, we induced postprandial inflammation by administrating a high-saturated-fat, high-carbohydrate test meal (∼1000 kcal, 33% kcal from saturated fat and 36% kcal from carbohydrate) that included coconut chicken curry, a corn muffin, and a cinnamon biscuit, and assessed plasma inflammatory cytokine concentration and inflammatory cytokine secretion from LPS-stimulated PBMCs after consumption of the test meal containing 0 g, 2 g, and 6 g spice blend in a crossover design.

In optimal physiologic (healthy) conditions, postprandial inflammation is adequately controlled by a variety of nutrient-sensing regulatory mechanisms, making it challenging to capture the therapeutic effect of nutritional interventions on postprandial inflammation. In contrast, subjects at risk of metabolic syndrome have a higher systemic stress response to high-fat challenges than do healthy subjects (43). Given the exaggerated postprandial inflammatory response in this population with elevated systemic inflammatory tone, it is often easier to detect the effectiveness of therapeutic nutritional interventions. The participants recruited for the current study were men with overweight/obesity who also had an elevated waist circumference and had ≥1 other CVD risk factor (elevated LDL cholesterol and TGs, reduced HDL cholesterol, elevated CRP, elevated blood pressure, or elevated fasting glucose). Therefore, these participants were expected to have chronic low-grade inflammation with prolonged, increased oxidative and inflammatory stress in response to the HFCM challenge. We hypothesized that spice consumption in conjunction with the HFCM would reduce postprandial inflammation in this population with elevated inflammatory mediators.

Monocytes are the main inflammatory cell type that invade the arterial wall during the development of atherosclerosis, and their subsequent differentiation into macrophages and the formation of foam cells are implicated in all stages of atherosclerotic lesion formation (44). Postprandial hyperlipidemia after a high-fat challenge is accompanied by a transient increase in monocyte number with concomitant production of proinflammatory cytokines, which may contribute to endothelial dysfunction (45–47). Consistent with previous studies, we demonstrated a significant increase in the percentage of monocytes (CD14^+^/HLA-DR^+^) after HFCM consumption. Schildberger et al. (48) report that PBMCs and monocytes secrete comparable amounts of IL-1β, IL-6, IL-8, and TNF-α after LPS stimulation, suggesting that the monocyte subset in PBMCs is primarily responsible for the production of inflammatory cytokines. We, therefore, adjusted cytokine secretion from LPS-stimulated PBMCs by CD14^+^/HLA-DR^+^ monocyte number to determine cytokine secretion per monocyte.

Glucose was included as a covariate in the model based on data collected in previous studies, and the association observed between change in plasma glucose and cytokine secretion in our model. In a cross-sectional study including subjects aged 35–75 y (*n* = 5176) living in Lausanne, Switzerland, a positive correlation between plasma cytokines (IL-6 and TNF-α) and fasting plasma glucose was observed (49). In addition, proinflammatory cytokine secretion from LPS-stimulated PBMCs is significantly elevated after the pretreatment of PBMCs with glucose compared with no pretreatment (50), suggesting that glucose modulates cytokine secretion from LPS-stimulated PBMCs. In the current study, adding change in plasma glucose to the mixed-effect model significantly changed plasma cytokine concentration and cytokine secretion from LPS-stimulated PBMCs after consumption of the HFCM without spices, demonstrating that the variability in plasma glucose after consumption of the HFCM may be influencing cytokine response to the meal.

Our data on cytokine secretion from LPS-stimulated PBMCs after consumption of the HFCM suggest that the pattern of secretion in response to this stimulus may be altered by consumption of the HFCM containing the 6 g spice blend. IL-1β and IL-8 secretion at 180 min, and IL-6 and IL-8 secretion at 240 min after HFCM consumption containing the 6 g spice blend were significantly lower than the 2 g spice blend. Consumption of the 6 g spice blend significantly reduced IL-β secretion at 240 min after HFCM consumption compared with the 0 g spice blend. Lastly, the change in IL-6, IL-8, MCP-1, and TNF-α secretion from LPS-stimulated PBMCs at 240 min after HFCM consumption containing the 6 g spice blend, was below baseline values for each cytokine, suggesting that the HFCM-induced increase in inflammatory cytokine secretion may be blunted by the consumption of an HFCM containing 6 g spice blend.

Although a dose–response relation between phytochemical consumption and chronic disease risk has been reported (51), we only observed a dose-response effect of the spice blend on IL-1β secretion. It is possible that there is a dose threshold between 2 and 6 g spice blend below which the anti-inflammatory effect of spice consumption does not occur. However, additional studies are needed to test this hypothesis. In contrast to the change in cytokine secretion from LPS-stimulated PBMCs after spice consumption with the HFCM, we observed no effect of spice consumption on plasma cytokine concentrations. Herieka and Erridge (52) demonstrate that whereas elevated postprandial inflammatory markers are reliably detected in leukocytes (either extracellularly or intracellularly), they are not consistently raised in plasma. Our data support these findings, and suggest that LPS stimulation may be a more sensitive way to assess monocyte function because we are directly quantifying the cytokine response to LPS stimulation by monocytes in the PBMC compartment rather than measuring the accumulation of cytokines secreted from various cell sources in the circulation.

Obesity-induced, chronic, low-grade inflammation results from activation of several inflammatory signaling cascades, which includes the NF-κB pathway. NF-κB is a key mediator of inflammation because it regulates a large array of genes encoding proinflammatory cytokines (53). Accumulating evidence suggests that the bioactive metabolites of spices can prevent chronic inflammation by targeting the NF-κB signaling pathway (20). Turmeric, ginger, and cinnamon were the spices that comprised the highest proportion of the spice blend in our study. Numerous *in vitro* and *in vivo* animal and human studies have demonstrated anti-inflammatory effects of these spices. In randomized controlled trials, curcumin supplementation reduced serum IL-1β and IL-4 in adults with obesity (1 g/d for 8 wk) (33) and reduced serum IL-6, MCP-1, and TNF-α concentrations in subjects with metabolic syndrome (1 g/d for 4 wk) (34). In diabetic rats, curcumin reduced circulating IL-6, MCP-1, and TNF-α concentrations (100 mg · kg body weight^–1^ · d^–1^ for 7 wk) (54). *In vitro* studies have demonstrated that curcumin treatment suppresses IL-6 and TNF-α secretion after the inhibition of the NF-κB activity from LPS-stimulated RAW264.7 cells (55, 56) and LPS-stimulated THP-1 cells (57). Ginger supplementation reduced CRP in subjects with type 2 diabetes (1.6–3 g/d for 12 wk) (35, 36) and nonalcoholic fatty liver disease (2 g/d for 12 wk) (37). In mice with LPS-induced acute systemic inflammation, supplementation of ginger reduced serum IFN-γ and IL-6 (100–1000 mg · kg body weight^–1^ · d^–1^ for 3 d) (58). The mechanism underlying the anti-inflammatory effect of ginger is likely the suppression of NF-κB activity by the bioactive 6-gingerol. 6-Gingerol inhibited the LPS-stimulated NF-κB activity in murine peritoneal macrophages, resulting in decreased IL-1β secretion from the macrophages (59). Similarly, oral administration of cinnamon extract significantly reduced serum TNF-α after LPS stimulation in BALB/c mice (20–500 mg · kg body weight^–1^ · d^–1^ for 6 d) (60). Preincubation of THP-1 human monocytes with cinnamon extract significantly reduced LPS-stimulated IL-8 secretion (61). *In vitro* studies have reported that cinnamaldehyde inhibits NF-κB activity in RAW264.7 murine macrophages (40, 62, 63), suggesting the anti-inflammatory property of cinnamon may be due to cinnamaldehyde, a major bioactive compound from cinnamon bark. Moreover, other spices and their bioactive compounds, such as basil (estragole) (64, 65), black pepper (piperine) (66), coriander (linalool) (67), cumin (cuminaldehyde) (68), red pepper (capsaicin) (69, 70), rosemary (rosmarinic acid) (71), and thyme (thymol) (72), may also have anti-inflammatory effects. Overall, based on previous studies, the reduction in IL-1β, IL-6, and IL-8 secretion from LPS-stimulated PBMCs after the meal challenge in the current study may be mediated via spice-induced inhibition of NF-κB activity, which should be explored in future studies.

A major strength of our study is the study design (3-period, crossover, randomized controlled trial) because each participant served as his own control. Another strength of the study is that we quantified the LPS-stimulated cytokine production from PBMCs, which enabled us to evaluate monocyte function in subjects after a meal with and without the addition of the spice blend. Limitations of this study are the small sample size (*n* = 12) and homogeneity of the participants. In addition, because this was not a double-blinded study (i.e., participants could differentiate between spiced and nonspiced meals), a potential bias may exist because subjects were aware of their treatment group. However, the outcomes we evaluated in this study are objective markers (i.e., inflammatory cytokine concentrations) measured in blood or in culture supernatants, thus the potential for bias may be small. In addition, the inflammatory mediators evaluated in this study are secondary outcomes; therefore, adjustment for multiple outcomes is not necessary for these data. However, because analysis of multiple outcomes for the same study inflates the false positive rate, the results should be viewed with caution. To ensure the findings from the current study are broadly applicable, the results from the current study need to be replicated in a larger, more diverse population. Moreover, it would be interesting to explore the potential mechanisms underlying the anti-inflammatory effect of spices, such as nuclear translocation and activity of NF-κB in PBMCs, in future studies.

In summary, we demonstrated that the presence of the 6 g spice blend in a HFCM significantly reduced postprandial IL-1β, IL-6, and IL-8 secretion from LPS-stimulated PBMCs in men with overweight/obesity at risk of CVD, suggesting a potential anti-inflammatory effect of spices. These findings are the first to our knowledge to demonstrate that consumption of a HFCM containing a spice blend could modulate postprandial cytokine secretion from culture PBMCs, suggesting the incorporation of spices into daily diet may help reduce postprandial inflammation and concurrently attenuate chronic low-grade inflammation.

## Supplementary Material

nxaa063_Supplemental_Tables_and_Figure

## Notes

Supported by McCormick Science Institute MSI-10001 (to PMK-E and CJR), NIH TL1 training program TL1TR002016 (to ESO), and National Center for Advancing Translational Sciences at the NIH grant 1UL1TR002014-01.

Author disclosures: KSP and PMK-E received a travel reimbursement and an honorarium from McCormick Science Institute to discuss the study findings with a regulatory agency. The other authors report no conflicts of interest.

Supplemental Tables 1–4 and Supplemental Figure 1 are available from the “Supplementary data” link in the online posting of the article and from the same link in the online table of contents at https://academic.oup.com/jn/.

Abbreviations used: CRP, C-reactive protein; CVD, cardiovascular disease; DBP, diastolic blood pressure; HFCM, high-saturated-fat, high-carbohydrate meal; Human Leukocyte Antigen-DR isotype, HLA-DR; MCP, monocyte chemoattractant protein; PBMC, peripheral blood mononuclear cell; ROS, reactive oxygen species; SBP, systolic blood pressure; TCA, tricarboxylic acid; TG, triglyceride.
