# Supplementary material for: Spices in a High-Saturated-Fat, High-Carbohydrate Meal Reduce Postprandial Proinflammatory Cytokine Secretion in Men with Overweight or Obesity: A 3-Period, Crossover, Randomized Controlled Trial
Source: J Nutr. 2020 Mar 25;150(6):1600–9. doi: 10.1093/jn/nxaa063 (PMC7269750; doi:10.1093/jn/nxaa063)
Supplement: nxaa063_Supplemental_Tables_and_Figure [file nxaa063_supplemental_tables_and_figure.docx]

**Supplemental Table 1. SAS codes for statistical analysis**

|  | **SAS code** |
| --- | --- |
| **Percent of monocytes, T cells, B cells and NK cells)** | PROC MIXED DATA=RAWDATA;  class Subject Spice Time;  model Mon=Spice Time Spice*Time/residual;  RANDOM Subject;  REPEATED Time*Spice /type=AR(1) subject=Subject rcorr;  RUN; |
| **Plasma or supernatant cytokines (only plasma IL-1β is shown, same for the other cytokines)** | PROC MIXED DATA=RAWDATA;  class Subject Spice Time;  model IL1b_plasma_change=Time Spice Spice*Time  Glucose_change Spice*Glucose_change/residual;  RANDOM Subject;  REPEATED Spice*Time /type=AR(1) subject=Subject;  RUN; |
| **Supernatant cytokines for each dose (only plasma IL-1β is shown, same for the other cytokines)** | PROC MIXED DATA=RAWDATA;  class Subject Time;  model IL1b_sups_change=Time/residual;  RANDOM Subject;  REPEATED Time/type=AR(1) subject=Subject;  LSMEANS Time/pdiff adjust=Tukey;  RUN; |
| **Supernatant cytokines at each time point (only plasma IL-1β is shown, same for the other cytokines)** | PROC MIXED DATA=RAWDATA;  class Subject Spice;  model IL1b_sups_change=Spice/residual;  RANDOM Subject;  REPEATED Spice/type=AR(1) subject=Subject;  LSMEANS Spice/pdiff adjust=Tukey;  RUN; |

**Supplemental Table 2. The effect of time on change (∆C) in cytokine secretion from LPS-stimulated PBMCs in each treatment group after high-saturated fat, high-carbohydrate meal (HFCM) consumption in men with overweight or obesity**

|  | **ΔC_0min_** | **ΔC_60min_** | **ΔC_120min_** | **ΔC_180min_** | **ΔC_240min_** | **Time**  **ANOVA**  ***P*-value** |
| --- | --- | --- | --- | --- | --- | --- |
| **IL-1β (ng/mL)** |  |  |  |  |  |  |
| HFCM | 0^b^ | 11.12 ± 4.63^a^ | 3.93 ± 3.32^b^ | 0.93 ± 2.20^b^ | 1.59 ± 3.46^b^ | **<0.01** |
| HFCM + 2 g | 0^a^ | 7.39 ± 9.12^a^ | 6.51 ± 8.08^a^ | 19.57 ± 4.73^a^ | 0.70 ± 4.69^b^ | **0.02** |
| HFCM + 6 g | 0 | -4.79 ± 10.49 | -2.18 ± 8.94 | -13.88 ± 8.80 | -19.33 ± 7.98 | 0.33 |
| **IL-6 (ng/mL)** |  |  |  |  |  |  |
| HFCM | 0 | 48.33 ± 26.56 | 16.68 ± 22.46 | -1.46 ± 13.73 | 8.97 ± 19.65 | 0.16 |
| HFCM + 2 g | 0 | 20.84 ± 35.34 | -5.92 ± 29.20 | 71.46 ± 34.96 | 11.53 ± 42.82 | 0.36 |
| HFCM + 6 g | 0 | -17.33 ± 38.30 | 14.27 ± 30.83 | -22.59 ± 23.75 | -39.77 ± 22.57 | 0.35 |
| **IL-8 (ng/mL)** |  |  |  |  |  |  |
| HFCM | 0 | 192.95 ± 94.10 | 67.26 ± 85.15 | -122.12 ± 51.99 | 26.38 ± 60.50 | 0.07 |
| HFCM + 2 g | 0 | 132.08 ± 89.87 | 57.79 ± 90.45 | 228.00 ± 81.73 | 30.30 ± 145.49 | 0.33 |
| HFCM + 6 g | 0 | -26.81 ± 133.90 | 69.22 ± 160.30 | -124.42 ± 106.19 | -220.80 ± 89.99 | 0.23 |
| **MCP-1 (ng/mL)** |  |  |  |  |  |  |
| HFCM | 0 | 3.47 ± 2.59 | -2.31 ± 2.13 | -0.97 ± 4.14 | -2.61 ± 4.32 | 0.33 |
| HFCM + 2 g | 0 | 4.08 ± 5.89 | 1.16 ± 3.49 | 5.37 ± 5.80 | -4.06 ± 2.94 | 0.35 |
| HFCM + 6 g | 0 | -4.66 ± 5.64 | -1.33 ± 4.69 | 0.11 ± 4.23 | -10.75 ± 6.29 | 0.22 |
| **TNF-α (ng/mL)** |  |  |  |  |  |  |
| HFCM | 0 | 13.05 ± 7.47 | 7.88 ± 11.72 | 2.82 ± 8.91 | 4.27 ± 11.32 | 0.75 |
| HFCM + 2 g | 0 | 17.32 ± 9.68 | -2.92 ± 21.66 | 57.90 ± 19.08 | -4.29 ± 28.65 | 0.08 |
| HFCM + 6 g | 0 | -18.91 ± 22.84 | 14.56 ± 23.33 | -7.21 ± 21.22 | -25.06 ± 16.93 | 0.24 |

Data are presented as mean ± SEM, n=12

Labeled means at each time point without a common letter differ, *P* < 0.05.

**Supplemental Table 3. Plasma cytokine concentration after high-saturated fat, high-carbohydrate meal (HFCM) consumption in men with overweight or obesity at risk for cardiovascular disease***

| **Cytokine**  **(pg/mL)** | **Time after meal (min)** | **HFCM** | **HFCM + 2g spice blend** | **HFCM + 6g spice blend** | **Time**  ***P-*value** | **Spice**  ***P-*value** | **Time**  $\boldsymbol{\times}$  **Spice**  ***P-*value** |
| --- | --- | --- | --- | --- | --- | --- | --- |
| **IFN-γ** | **0** | 5.83 ± 2.70 | 7.26 ± 2.63 | 5.99 ± 2.66 | 0.12 | **<0.001** | 0.07 |
|  | **60** | 5.96 ± 2.70 | 6.60 ± 2.40 | 5.57 ± 2.57 |  |  |  |
|  | **120** | 5.99 ± 2.67 | 6.58 ± 2.41 | 5.80 ± 2.65 |  |  |  |
|  | **180** | 6.04 ± 2.65 | 6.68 ± 2.39 | 5.78 ± 2.60 |  |  |  |
|  | **240** | 6.03 ± 2.61 | 6.40 ± 2.31 | 5.79 ± 2.65 |  |  |  |
| **IL-2** | **0** | 0.66 ± 0.29 | 0.70 ± 0.28 | 0.70 ± 0.28 | **<0.001** | **<0.001** | **<0.001** |
|  | **60** | 0.67 ± 0.28 | 0.70 ± 0.29 | 0.65 ± 0.27 |  |  |  |
|  | **120** | 0.69 ± 0.28 | 0.70 ± 0.29 | 0.66 ± 0.27 |  |  |  |
|  | **180** | 0.69 ± 0.27 | 0.67 ± 0.27 | 0.67 ± 0.28 |  |  |  |
|  | **240** | 0.70 ± 0.28 | 0.64 ± 0.27 | 0.66 ± 0.27 |  |  |  |
| **IL-4** | **0** | 0.12 ± 0.06 | 0.14 ± 0.06 | 0.12 ± 0.06 | 0.47 | **0.043** | 0.77 |
|  | **60** | 0.13 ± 0.06 | 0.13 ± 0.05 | 0.12 ± 0.06 |  |  |  |
|  | **120** | 0.14 ± 0.06 | 0.13 ± 0.05 | 0.13 ± 0.06 |  |  |  |
|  | **180** | 0.13 ± 0.06 | 0.13 ± 0.05 | 0.13 ± 0.05 |  |  |  |
|  | **240** | 0.14 ± 0.05 | 0.13 ± 0.05 | 0.13 ± 0.06 |  |  |  |
| **IL-10** | **0** | 0.52 ± 0.25 | 0.63 ± 0.26 | 0.53 ± 0.23 | 0.98 | **0.034** | 0.34 |
|  | **60** | 0.53 ± 0.23 | 0.60 ± 0.25 | 0.53 ± 0.23 |  |  |  |
|  | **120** | 0.55 ± 0.24 | 0.60 ± 0.25 | 0.52 ± 0.22 |  |  |  |
|  | **180** | 0.53 ± 0.23 | 0.60 ± 0.26 | 0.55 ± 0.24 |  |  |  |
|  | **240** | 0.54 ± 0.24 | 0.60 ± 0.26 | 0.52 ± 0.23 |  |  |  |
| **IL-12p70** | **0** | 0.50 ± 0.25 | 0.54 ± 0.26 | 0.50 ± 0.24 | 0.83 | 0.07 | 0.47 |
|  | **60** | 0.52 ± 0.24 | 0.51 ± 0.24 | 0.48 ± 0.24 |  |  |  |
|  | **120** | 0.52 ± 0.24 | 0.52 ± 0.24 | 0.51 ± 0.24 |  |  |  |
|  | **180** | 0.52 ± 0.24 | 0.52 ± 0.24 | 0.51 ± 0.24 |  |  |  |
|  | **240** | 0.52 ± 0.25 | 0.51 ± 0.23 | 0.53 ± 0.25 |  |  |  |
| **IL-13** | **0** | 5.91 ± 1.56 | 6.11 ± 1.59 | 5.78 ± 1.47 | 0.47 | 0.31 | 0.89 |
|  | **60** | 5.90 ± 1.55 | 6.05 ± 1.55 | 5.83 ± 1.51 |  |  |  |
|  | **120** | 5.94 ± 1.53 | 6.01 ± 1.55 | 5.82 ± 1.50 |  |  |  |
|  | **180** | 5.96 ± 1.53 | 6.06 ± 1.55 | 5.90 ± 1.50 |  |  |  |
|  | **240** | 5.86 ± 1.51 | 6.02 ± 1.53 | 5.83 ± 1.50 |  |  |  |

* Data are presented as mean ± SEM, n=12

**Supplemental Table 4. Cytokine secretion from LPS-stimulated PBMCs after high-saturated fat, high-carbohydrate meal (HFCM) consumption in men with overweight or obesity***

| **Cytokine**  **(pg/mL)** | **Time after meal (min)** | **HFCM** | **HFCM + 2g spice blend** | **HFCM + 6g spice blend** | **Time**  ***P-*value** | **Spice**  ***P-*value** | **Time**  $\boldsymbol{\times}$  **Spice**  ***P-*value** |
| --- | --- | --- | --- | --- | --- | --- | --- |
| **IFN-γ** | **0** | 4.91 ± 1.25 | 2.32 ± 0.96 | 1.99 ± 0.84 | 0.44 | 0.15 | 0.35 |
|  | **60** | 3.63 ± 1.18 | 4.83 ± 1.09 | 3.37 ± 1.08 |  |  |  |
|  | **120** | 2.83 ± 0.70 | 3.24 ± 0.94 | 9.81 ± 3.68 |  |  |  |
|  | **180** | 2.82 ± 0.81 | 7.31 ± 2.23 | 9.67 ± 5.41 |  |  |  |
|  | **240** | 4.27 ± 1.01 | 5.98 ± 1.51 | 6.00 ± 2.13 |  |  |  |
| **IL-2** | **0** | 0.99 ± 0.39 | 1.04 ± 0.22 | 1.08 ± 0.33 | 0.40 | **0.040** | 0.10 |
|  | **60** | 1.31 ± 0.47 | 1.07 ± 0.25 | 0.58 ± 0.21 |  |  |  |
|  | **120** | - 1. ± 0.21 | 0.81 ± 0.28 | 1.57 ± 0.33 |  |  |  |
|  | **180** | 1.00 ± 0.34 | 1.20 ± 0.33 | 1.40 ± 0.39 |  |  |  |
|  | **240** | 1.43 ± 0.42 | 1.01 ± 0.33 | 1.63 ± 0.39 |  |  |  |
| **IL-4** | **0** | 2.03 ± 0.43 | 2.05 ± 0.27 | 1.38 ± 0.19 | 0.16 | 0.15 | 0.51 |
|  | **60** | 2.20 ± 0.57 | 2.08 ± 0.30 | 1.48 ± 0.18 |  |  |  |
|  | **120** | 2.08 ± 0.32 | 2.23 ± 0.41 | 1.95 ± 0.20 |  |  |  |
|  | **180** | 2.20 ± 0.37 | 1.76 ± 0.29 | 1.87 ± 0.24 |  |  |  |
|  | **240** | 2.21 ± 0.30 | 2.02 ± 0.30 | 2.43 ± 0.38 |  |  |  |
| **IL-10** | **0** | 6.82 ± 1.41 | 6.24 ± 1.53 | 4.10 ± 1.07 | 0.35 | 0.17 | 0.17 |
|  | **60** | 6.83 ± 1.19 | 4.69 ± 1.20 | 6.02 ± 1.28 |  |  |  |
|  | **120** | 6.39 ± 1.21 | 7.10 ± 1.59 | 8.17 ± 1.67 |  |  |  |
|  | **180** | 6.50 ± 1.58 | 7.90 ± 2.04 | 7.91 ± 1.42 |  |  |  |
|  | **240** | 7.44 ± 1.52 | 7.20 ± 1.72 | 6.93 ± 1.41 |  |  |  |
| **IL-12p70** | **0** | 2.53 ± 0.60 | 1.80 ± 0.42 | 1.38 ± 0.40 | 0.63 | 0.75 | 0.33 |
|  | **60** | 2.50 ± 0.44 | 2.25 ± 0.55 | 1.93 ± 0.53 |  |  |  |
|  | **120** | 1.86 ± 0.35 | 2.32 ± 0.63 | 2.78 ± 0.60 |  |  |  |
|  | **180** | 2.18 ± 0.54 | 2.17 ± 0.76 | 2.45 ± 0.55 |  |  |  |
|  | **240** | 2.93 ± 0.70 | 2.41 ± 0.71 | 2.41 ± 0.70 |  |  |  |

* Data are presented as mean ± SEM, n=12

**Supplemental Figure 1**

**
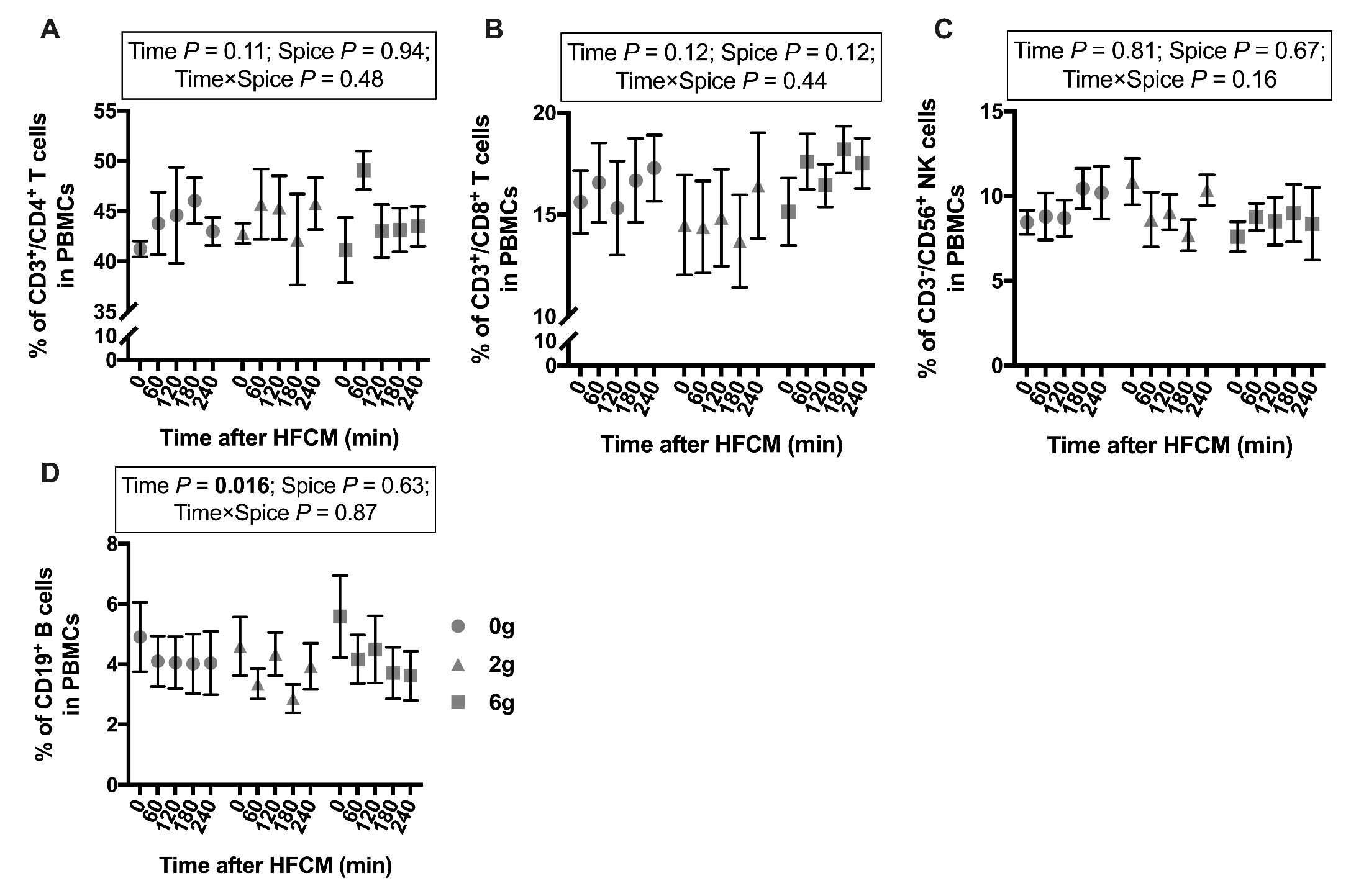
**

**Supplemental Figure 1.** The percent of (A) CD3^+^/CD4^+^ T cells, (B) CD3^+^/CD8^+^ T cells, (C) CD3^-^/CD56^+^ NK cells and (D) CD19^+^ B cells in circulation after a high-saturated fat, high-carbohydrate meal (HFCM) challenge in men with overweight or obesity at risk for cardiovascular disease. Mean ± SEM, n=7.
